# Hypermethylation of tumor suppressor lncRNA *MEF2C-AS1* frequently happened in patients at all stages of colorectal carcinogenesis

**DOI:** 10.1186/s13148-022-01328-1

**Published:** 2022-09-05

**Authors:** Sangni Qian, Shujuan Lin, Xin Xu, Hao Bai, Aibuta Yeerken, Xiaojiang Ying, Zhenjun Li, Xinglin Fei, Jinhua Yang, Mengling Tang, Jianbing Wang, Mingjuan Jin, Kun Chen

**Affiliations:** 1 Department of Public Health, Second Affiliated Hospital, Zhejiang University School of Medicine, Hangzhou, 310058 China; 2 Department of Public Health, Hangzhou First People’s Hospital, Zhejiang University School of Medicine, Hangzhou, 310058 China; 3 Department of Anorectal Surgery, Shaoxing People’s Hospital, Shaoxing, 312000 China; 4 Jiashan Institute of Cancer Prevention and Treatment, Jiaxing, 314100 China; 5 Department of Public Health, Fourth Affiliated Hospital, Zhejiang University School of Medicine, Hangzhou, 310058 China; 6 Department of Public Health, National Clinical Research Center for Child Health of the Children’s Hospital, Zhejiang University School of Medicine, Hangzhou, 310058 China

**Keywords:** Colorectal cancer, DNA methylation, *MEF2C-AS1*, Carcinogenesis, Expression

## Abstract

**Background:**

The novel long noncoding RNA *MEF2C-AS1* has been identified to play suppressor roles during tumorigenesis. DNA methylation has a regulatory effect on gene expression in cancer initiation and progression. However, the methylation status of *MEF2C-AS1* and its role in colorectal cancer (CRC) development remain unclear.

**Methods:**

The expression and methylation levels of *MEF2C-AS1* were systematically analyzed among 31 cancers with available qualified data in GEPIA and UCSC Xena databases. Then, the *MEF2C-AS1* methylation status was firstly examined among 12 CRCs by Illumina Infinium MethylationEPIC BeadChip in in-house step 1 and further quantified among 48 CRCs by the MassARRAY method in in-house step 2. Subsequently, its methylation and expression levels were quantified among 81 non-advanced adenomas (NAAs), 81 advanced adenomas (AAs), and 286 CRCs using the MassARRAY method, and among 34 NAAs, 45 AAs, and 75 CRCs by qRT-PCR, in in-house step 3, respectively. The effect of *MEF2C-AS1* methylation on CRC survival was analyzed by the Kaplan–Meier method. Additionally, in vitro cell proliferation, migration and invasion assays, and bioinformatics analysis were performed to explore the role of *MEF2C-AS1* in colorectal carcinogenesis.

**Results:**

Lower expression and higher methylation of *MEF2C-AS1* were found in CRC by online databases. In the comparisons of lesion tissues with adjacent normal tissues, *MEF2C-AS1* hypermethylation of each individual site and mean level was found among CRC patients in in-house step 1 and step 2, more meaningfully, among NAA patients, AA patients, and CRC patients at all stages during colorectal carcinogenesis in in-house step 3 (all *p* < 0.05). Further comparisons demonstrated significant differences between CRC and NAA (*p* = 0.025), AA and NAA (*p* = 0.020). Moreover, *MEF2C-AS1* hypermethylation was associated with poorer disease-specific survival of CRC patients (*p* = 0.044). In addition, hypermethylation and lower expression of *MEF2C-AS1* were verified in RKO cells, and the *MEF2C-AS1* overexpression significantly suppressed RKO cell proliferation, migration, and invasion.

**Conclusions:**

The findings reveal that *MEF2C-AS1* hypermethylation might be an early driven event during colorectal carcinogenesis. It might serve as a promising prognostic biomarker for CRC survival. Our study also indicates the potential tumor-suppressing role of *MEF2C-AS1* in CRC.

**Supplementary Information:**

The online version contains supplementary material available at 10.1186/s13148-022-01328-1.

## Background

Colorectal cancer (CRC) is the third most commonly diagnosed malignancy and the second leading cause of cancer death worldwide [1]. Most sporadic CRCs are recognized to develop from colorectal adenomas following the adenoma–carcinoma sequence [2]. In the complex multi-step process of colorectal carcinogenesis and progression, environmental factors, genetic factors, and epigenetic factors play important roles in composing a regulatory network involving many molecules [3–5].

Long noncoding RNAs (lncRNAs) are defined as transcripts of more than 200 nucleotides that are not translated into proteins except for functional small peptides [6]. Nowadays, these transcripts are involved in various diseases related to biological behaviors such as cell proliferation, differentiation, and migration [7–9], and their aberrant expression is significantly associated with the initiation and development of cancers [10]. For instance, an oncogenic lncRNA *BLACAT1* was reported in osteosarcoma (OS) [11], pancreatic cancer (PC) [12], breast cancer (BC) [13], and CRC [14]. The elevated expression of *BLACAT1 *was detected in lesion tissues and was associated with poor prognosis of patients with cervical squamous cell carcinoma (CSCC) [15] and CRC [14]. Until now, plenty of functional lncRNAs have been characterized and they might regulate target mRNAs expression through the competitive endogenous RNA (ceRNA) mechanism mediated by miRNAs [16]. Nevertheless, the potential molecules participating in the specific regulatory network need to be further explored.

Aberrant DNA methylation, expressed as the hypermethylation or hypomethylation of cytosine–guanine dinucleotide (CpG), has been proved to be one of the most important factors in regulating gene expression at the pretranscriptional level in human cancers [17, 18]. Specifically, tumor suppressor genes could be inhibited by their promoter hypermethylation and could be reactivated after demethylation; on the contrary, oncogenes might be activated by its hypomethylation [19–23]. For instance, hypermethylation and downregulation of lncRNA *LINC00472* were identified in CRC tissues as compared with adjacent normal tissues, and its hypermethylation might serve as a potential CRC diagnostic biomarker [24]. Recently, studies have revealed that methylation alteration could be detected at the early stages of colorectal carcinogenesis [25, 26]. Fan J et al. [26] found that aberrant hypermethylation of *ADHFE1* promoter had maintained throughout the low-grade adenoma and high-grade adenoma which were associated with colorectal adenoma development. However, the number of lncRNAs that have been reported to show aberrant methylation status during carcinogenesis was still limited.

A novel lncRNA gene *MEF2C* antisense RNA 1 (*MEF2C-AS1*), located at 5q14.3, has been identified to be downregulated and to play tumor suppressor roles in diffuse gastric cancer (DGC), cervical cancer (CC), and BC by inhibiting cell proliferation and aggressive tumor phenotypes [27–29]. However, the role of *MEF2C-AS1* in CRC development has not been investigated. Hence, this study aimed to clarify the *MEF2C-AS1* methylation status and its functional role during colorectal carcinogenesis. Firstly, we systematically analyzed *MEF2C-AS1* methylation and expression levels by online databases. Then, we validated the findings in our CRCs, and further among our additional non-advanced adenomas (NAAs), advanced adenomas (AAs), and CRCs to assess the *MEF2C-AS1* methylation status and expression changes at all stages of colorectal carcinogenesis. Furthermore, we examined the effect of *MEF2C-AS1* methylation on disease-specific survival (DSS) of CRC patients. Lastly, in vitro assays and bioinformatics analysis were performed to confirm the influence of *MEF2C-AS1* methylation on its expression and explore the role of *MEF2C-AS1* in colorectal carcinogenesis.

## Results

### The expression and DNA methylation status of *MEF2C-AS1* in various cancers by online databases

**Fig. 1:**
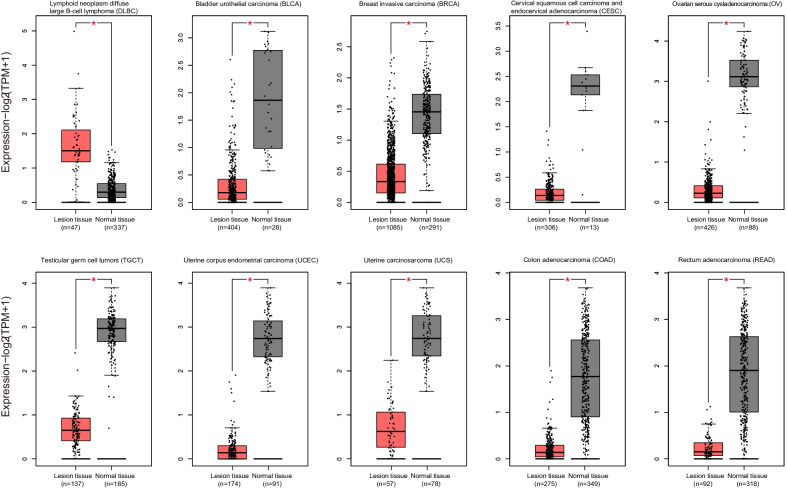
Differential *MEF2C-AS1* expression between lesion tissues and normal tissues among 10 cancers in the GEPIA database. Every dot represents the expression level for a tissue sample. Box plot in red or gray represents the distribution of expression level. Expression level is presented in log_2_(TPM + 1) scale. TPM: transcripts per million. *p < 0.05

**Fig. 2:**
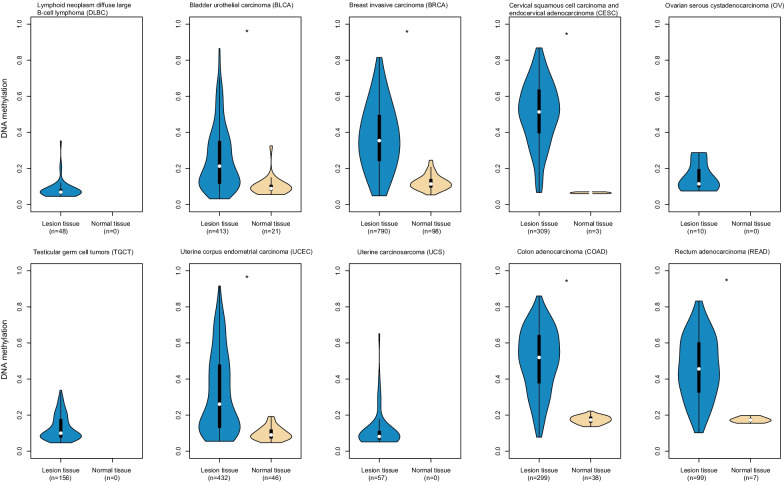
*MEF2C-AS1* hypermethylation among 10 cancers with differential expression in the UCSC Xena database. Violin plot in blue or orange represents the distribution of methylation level. *p < 0.05

*MEF2C-AS1* expression levels were compared between lesion tissues and normal tissues among 31 cancers with available RNA sequencing (RNA-Seq) data in the GEPIA database. Its expression levels were found to be significantly higher in lymphoid neoplasm diffuse large B cell lymphoma (DLBC), significantly lower in bladder urothelial carcinoma (BLCA), breast invasive carcinoma (BRCA), cervical squamous cell carcinoma and endocervical adenocarcinoma (CESC), ovarian serous cystadenocarcinoma (OV), testicular germ cell tumors (TGCT), uterine corpus endometrial carcinoma (UCEC), uterine carcinosarcoma (UCS), colon adenocarcinoma (COAD), and rectum adenocarcinoma (READ), but not significantly different in remaining 21 cancers, in the comparisons of lesion tissues and corresponding normal tissues (Fig. 1, Additional file 1: Fig. S1). To explore the relationship of *MEF2C-AS1* expression with its methylation, methylation levels were further compared between lesion tissues and normal tissues among 31 cancers if there were qualified methylation data from the UCSC Xena database. Among 10 cancers with differential *MEF2C-AS1* expression, higher methylation levels were found in lesion tissues of BLCA, BRCA, CESC, UCEC, COAD, and READ with available data (Fig. 2) compared with normal tissues, and comparisons of the remaining 21 cancers are presented in Additional file 1: Fig. S2. It is suggested that *MEF2C-AS1* might be downregulated and hypermethylated in CRC, which was further confirmed in the following study in consideration of sample availability.

### Primary comparison of *MEF2C-AS1* methylation status in in-house colorectal cancers

To clarify the methylation status of *MEF2C-AS1* in our samples, we quantified its promoter methylation levels among 12 CRC patients in in-house step 1 using Illumina MethylationEPIC BeadChip (850 K array). Specifically, the methylation levels of all 6 individual sites were found to be significantly higher in lesion tissues than those in adjacent normal tissues, and a similar difference was also observed in the comparison of their mean levels (Table 1, all *p* < 0.01).

**Table 1:** Differential analysis of *MEF2C-AS1* methylation among 12 CRC patients in in-house step 1

CpG site^a^	Location	Methylation level			*p* value^c^
		Lesion tissue	Adjacent normal tissue	Difference^b^	
cg04694437	chr5: 88,185,051	0.63	0.47	0.16	5.54E − 04
cg08966485	chr5: 88,185,090	0.66	0.37	0.29	2.69E − 07
cg15297153	chr5: 88,185,314	0.67	0.03	0.64	3.31E − 04
cg10571951	chr5: 88,185,387	0.75	0.06	0.69	5.11E − 08
cg12621171	chr5: 88,185,768	0.45	0.04	0.41	3.32E − 09
cg18109838	chr5: 88,185,987	0.43	0.07	0.36	2.23E − 03
Mean		0.60	0.17	0.43	2.15E − 07

*CRC*, colorectal cancer

^a^CpG site in the promoter region of *MEF2C-AS1*

^b^Methylation difference was calculated between lesion tissue and adjacent normal tissue

^c^Paired student’s *t* test

### Further validation of *MEF2C-AS1* hypermethylation in in-house colorectal cancers

**Fig. 3:**
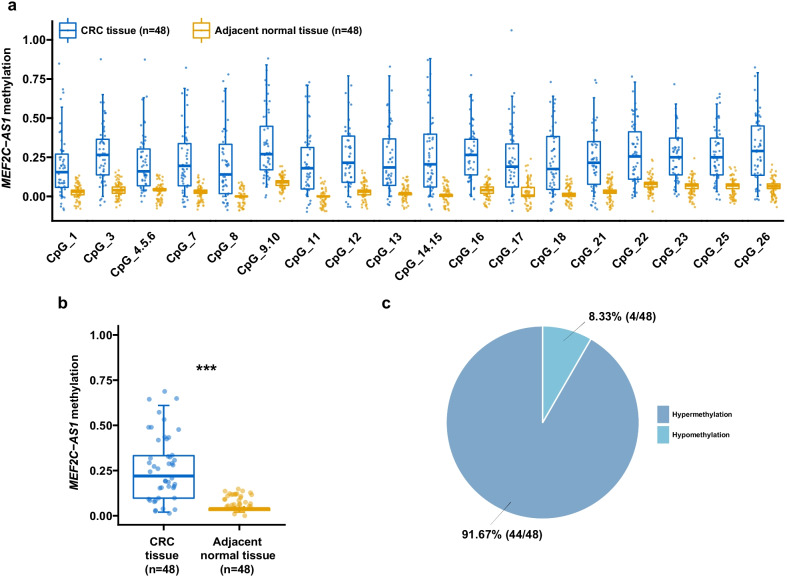
*MEF2C-AS1* hypermethylation among CRC patients in in-house step 2. **a** Methylation level of each individual CpG site. **b** Mean methylation level of all individual CpG sites. **c** Percentage of patients with hypermethylation or hypomethylation status. ***p < 0.001

The methylation status of *MEF2C-AS1* was reconfirmed among our additional 48 CRC patients in in-house step 2. By the MassARRAY method, methylation levels of 18 CpG sites were successfully detected and were then included in the subsequent analysis. As shown in Fig. 3a, all individual CpG sites were observed to be significantly hypermethylated in lesion tissues compared with adjacent normal tissues (all *p* < 0.001). The mean level of *MEF2C-AS1* methylation of lesion tissues was 0.25, which was remarkably higher than 0.04 of adjacent normal tissues (Fig. 3b, *p* < 0.001). *MEF2C-AS1* hypermethylation frequently happened in 91.67% (44/48) of CRC patients (Fig. 3c).

### Elucidation of *MEF2C-AS1* hypermethylation in in-house patients at all stages of colorectal carcinogenesis

**Fig. 4:**
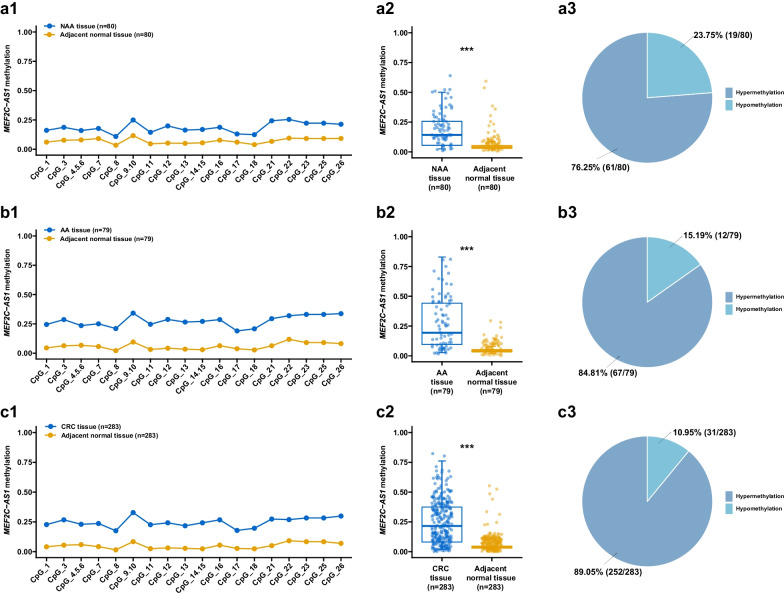
*MEF2C-AS1* hypermethylation among patients at all stages during colorectal carcinogenesis in in-house step 3. **a** NAA patients. **b** AA patients. **c** CRC patients. Line chart presents the methylation level of each individual CpG site. The distribution of the mean methylation level of all individual CpG sites is shown in the scatter plot. Pie chart presents the percentage of patients with hypermethylation or hypomethylation status. AA, advanced adenoma; CRC, colorectal cancer; NAA, non-advanced adenoma. ***p < 0.001

In in-house step 3, we ulteriorly quantified *MEF2C-AS1* methylation levels among 81 NAA, 81 AA, and 286 CRC patients by the MassARRAY method to elucidate its methylation status at all stages of colorectal carcinogenesis. In total, methylation levels were successfully measured for both lesion tissues and adjacent normal tissues in 80 NAAs, 79 AAs, and 283 CRCs. As expected, higher *MEF2C-AS1* methylation levels of individual CpG sites and mean levels were observed in lesion tissues of NAA (Fig. 4a, all *p* < 0.05), AA (Fig. 4b, all *p* < 0.001), and CRC (Fig. 4c, all *p* < 0.001) as compared with adjacent normal tissues, respectively. Compared with their adjacent normal tissues, the percentage of higher *MEF2C-AS1* methylation increased from 76.25% (61/80) of NAAs, to 84.81% (67/79) of AAs, and to 89.05% (252/283) of CRCs (Fig. 4).

Further comparisons of methylation in lesion tissues among patients are presented in Table 2. Interestingly, in most individual sites, significant differences were found between CRC and NAA, AA and NAA, but not between CRC and AA. The mean levels of *MEF2C-AS1* methylation in NAA, AA, and CRC tissues were 0.19, 0.28, and 0.25, respectively. Similarly, there were significant differences in comparisons of CRC with NAA (*p* = 0.025), AA with NAA (*p* = 0.020), but not in CRC with AA (*p* = 1.000).

**Table 2:** Comparisons of *MEF2C-AS1* methylation in lesion tissues from patients at different stages during colorectal carcinogenesis

CpG site	NAA		AA		CRC		*p* value^a^	AA vs. NAA		CRC vs. NAA		CRC vs. AA	
	*N*	*β*	*N*	*β*	*N*	*β*		Δ*β*	*p* value^b^	Δ*β*	*p* value^b^	Δ*β*	*p* value^b^
CpG_1	80	0.16	79	0.24	283	0.23	0.009	0.08	0.049	0.07	0.008	− 0.02	1.000
CpG_3	80	0.19	79	0.29	283	0.27	0.001	0.10	0.008	0.08	0.001	− 0.02	1.000
CpG_4.5.6	73	0.16	75	0.23	277	0.23	0.014	0.07	0.137	0.07	0.011	0.01	1.000
CpG_7	80	0.18	79	0.25	282	0.24	0.087	0.07	–	0.06	–	− 0.01	–
CpG_8	80	0.11	79	0.21	283	0.18	< 0.001	0.10	0.002	0.07	< 0.001	− 0.03	1.000
CpG_9.10	74	0.25	76	0.33	276	0.33	0.012	0.08	0.079	0.08	0.010	0.00	1.000
CpG_11	80	0.15	79	0.25	283	0.23	0.003	0.10	0.016	0.08	0.004	− 0.02	1.000
CpG_12	80	0.20	79	0.29	283	0.24	0.021	0.09	0.021	0.04	0.084	− 0.04	0.714
CpG_13	80	0.16	79	0.27	283	0.22	0.012	0.10	0.011	0.05	0.069	− 0.05	0.515
CpG_14.15	80	0.17	79	0.27	283	0.24	0.011	0.10	0.022	0.07	0.021	− 0.03	1.000
CpG_16	80	0.19	79	0.29	283	0.27	0.001	0.10	0.008	0.08	0.001	− 0.02	1.000
CpG_17	70	0.14	73	0.19	263	0.17	0.135	0.05	-	0.03	-	− 0.02	-
CpG_18	80	0.13	79	0.21	283	0.20	0.005	0.08	0.021	0.07	0.006	− 0.01	1.000
CpG_21	80	0.24	79	0.29	283	0.28	0.429	0.05	–	0.03	–	− 0.02	–
CpG_22	79	0.26	79	0.32	283	0.27	0.075	0.06	–	0.01	–	− 0.05	–
CpG_23	80	0.22	79	0.33	283	0.28	0.003	0.11	0.003	0.06	0.025	− 0.05	0.400
CpG_25	80	0.22	79	0.33	283	0.28	0.003	0.11	0.003	0.06	0.025	− 0.05	0.400
CpG_26	80	0.21	78	0.34	283	0.30	0.001	0.12	0.002	0.09	0.003	− 0.04	1.000
Mean	80	0.19	79	0.28	283	0.25	0.012	0.09	0.020	0.06	0.025	− 0.03	1.000

*p* < 0.05 was considered statistically significant

*AA*, advanced adenoma; *CRC*, colorectal cancer; *NAA*, non-advanced adenoma

^a^Kruskal–Wallis test

^b^Post hoc Dunn’s test

### Confirmation of low *MEF2C-AS1* expression in in-house patients at all stages of colorectal carcinogenesis

**Fig. 5:**
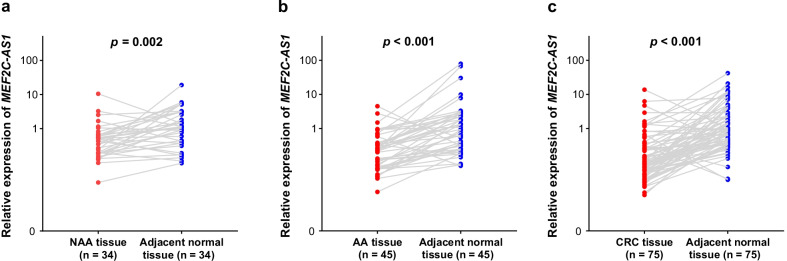
*MEF2C-AS1* expression among patients at all stages during colorectal carcinogenesis in in-house step 3. **a** NAA patients. **b** AA patients. **c** CRC patients. Each scatter in blue or red represents the expression level for a tissue sample. AA, advanced adenoma; CRC, colorectal cancer; NAA, non-advanced adenoma

To confirm the potential influence of *MEF2C-AS1* methylation on its expression, expression levels, detected by quantitative reverse transcription-polymerase chain reaction (qRT-PCR), were then compared between lesion tissues and adjacent normal tissues among 34 NAAs, 45 AAs, and 75 CRCs in in-house step 3. Significantly lower *MEF2C-AS1* expression was found in the comparisons of lesion tissues versus adjacent normal tissues of NAA patients (Fig. 5a, *p* = 0.002), AA patients (Fig. 5b, *p* < 0.001), and CRC patients (Fig. 5c, *p* < 0.001). However, additional comparisons of expression levels between lesion tissues showed significant lower expression as CRC compared with NAA (*p* < 0.001), but not as CRC compared with AA (*p* = 0.122), AA compared with NAA (*p* = 0.085).

### Effect of *MEF2C-AS1* methylation on disease-specific survival of colorectal cancer patients

**Fig. 6:**
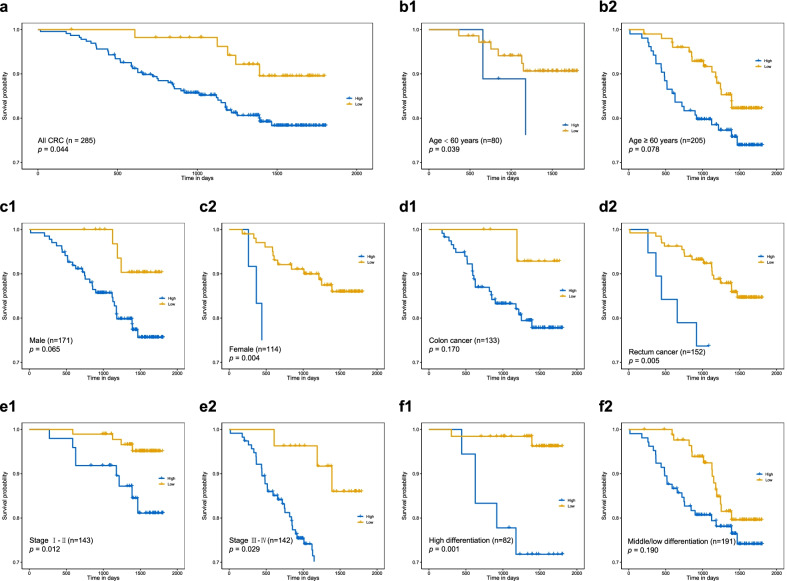
Survival analysis on CRC patients in in-house step 3. Kaplan–Meier method and log-rank test were used to compare DSS between CRC patients with high methylation and those with low methylation. **a** All CRC patients. Stratified and subgroup analyses were performed between the groups stratified by **b** age, **c** gender, **d** tumor location, **e** TNM stage, and **f** differentiation. CRC, colorectal cancer; DSS, disease-specific survival

Moreover, survival analysis was performed for CRC patients to examine the prognostic effect of the methylation. Among 285 CRC patients who successfully detected methylation status, 49 patients died of CRC during a median of 3.8 years of follow-up. The mean level of *MEF2C-AS1* methylation was negatively associated with DSS of CRC patients, indicating that patients with higher methylation had poorer survival than those with lower methylation (Fig. 6a, log-rank *p* = 0.044). In stratified and subgroup analyses, significant associations between *MEF2C-AS1* hypermethylation and poor DSS were observed in patients who were less than 60 years, female, and patients with rectum cancer or high differentiation cancer, and the associations were similar in patients with different stages (Fig. 6b–f).

### Influence of *MEF2C-AS1* methylation on its expression in colorectal cancer cells

**Fig. 7:**
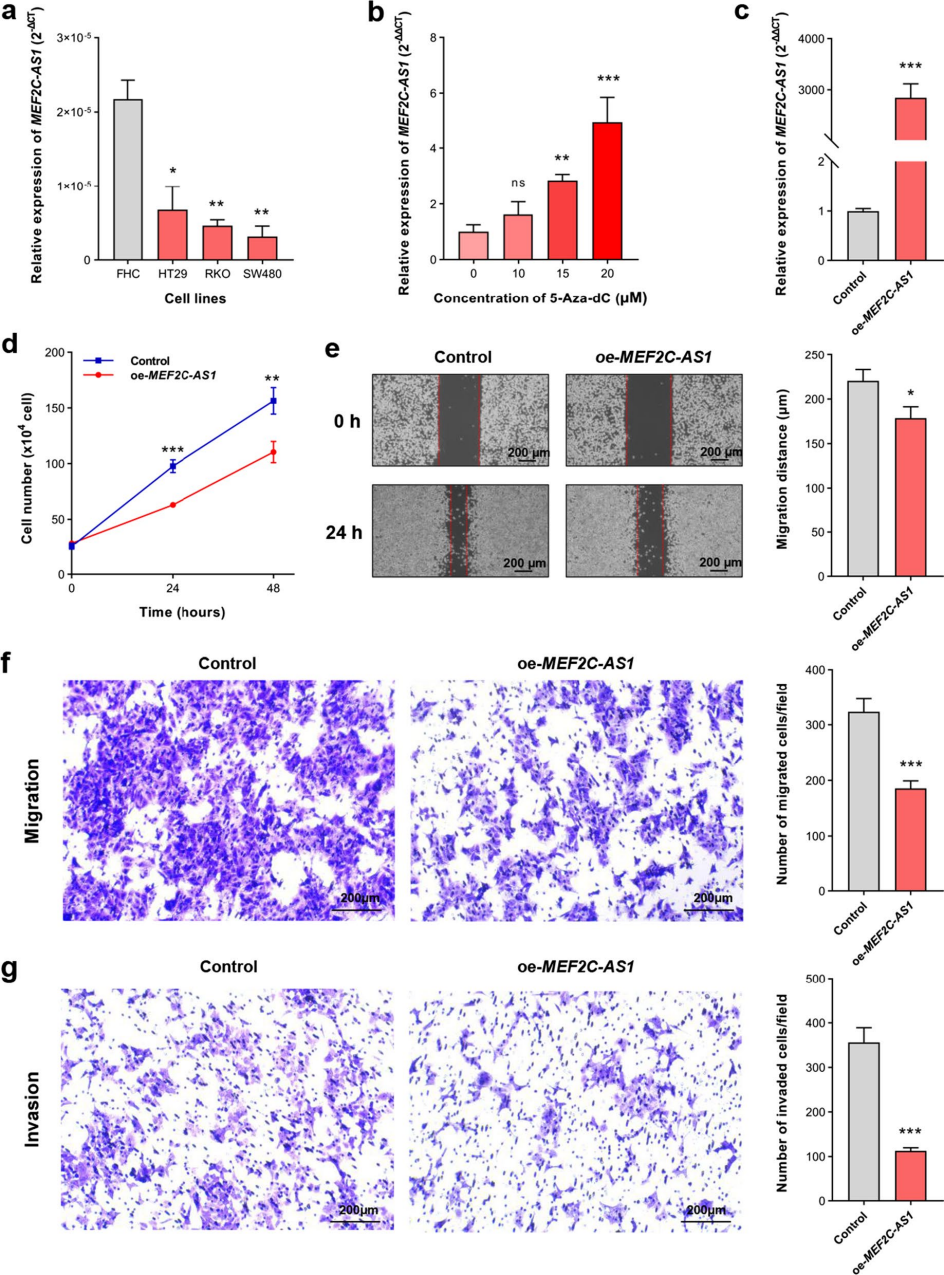
*MEF2C-AS1* expression levels in different cell lines and its functional role in cell proliferation, migration, and invasion in CRC cells. **a** The difference of *MEF2C-AS1* expression between normal colonic cell line and CRC cell lines. **b** *MEF2C-AS1* expression in RKO cells treated with different concentrations of demethylation agent 5-Aza-dC. **c** Transfection efficiency of *MEF2C-AS1* overexpression plasmid in RKO cells. The cells in the control group are treated with empty vectors. **d** Cell viability assay. **e** Wound healing assay. **f** Transwell migration assay. **g** Transwell invasion assay. 5-Aza-dC, 5-aza-2’-deoxycytidine; CRC, colorectal cancer. ***p < 0.001, **p < 0.01, *p < 0.05, ns *p* > 0.05

Based on the above findings of *MEF2C-AS1* methylation and expression in tissues, we further evaluated whether the expression of *MEF2C-AS1* was regulated by its aberrant promoter methylation in vitro. As shown in Fig. 7a, the decreased expression of *MEF2C-AS1* was found in all three CRC cell lines, HT29, RKO, and SW480, compared with normal colonic cell line FHC (all *p* < 0.05). After demethylation treatment with different concentrations of 5-aza-2'-deoxycytidine (5-Aza-dC) to the CRC cell line with moderate *MEF2C-AS1* downregulation, RKO cell line, its expression was significantly rescued in the 15 and 20 µM groups compared with the 0 µM group, and the effect showed a dose-dependent manner (Fig. 7b, all *p* < 0.05).

### Function of *MEF2C-AS1* on colorectal cancer cell proliferation, migration, and invasion

To determine the potential role of *MEF2C-AS1* in CRC, we performed several assays in RKO cells. Compared to cells transfected with an empty pLV3 vector, *MEF2C-AS1* expression levels significantly increased in cells transfected with a pLV3-MEF2C-AS1 plasmid (Fig. 7c). And cell proliferation was significantly inhibited in cells with *MEF2C-AS1* overexpression (Fig. 7d). Wound healing and transwell assays showed that *MEF2C-AS1* overexpression significantly reduced cell migration and invasion capacities (Fig. 7e–g) (all *p* < 0.05). To further explore the potential regulatory pathway, the putative target miRNAs and mRNAs of *MEF2C-AS1* were predicted by bioinformatics analysis using public databases. The comprehensive bioinformatics analysis showed that low expression of *MEF2C-AS1* in CRC might lead to downregulation of 10 target mRNAs including *KCNB1*, *CFL2*, *FAM129A*, *CLIP4*, *CYBRD1*, *SLC16A9*, *TMEM100*, *BVES*, *TRPM6*, and *ZEB1* through upregulating 3 miRNAs, hsa-miR-17-5p, hsa-miR-24-3p, and hsa-miR-429 (Additional file 1: Table S1).

## Discussion

Aberrant DNA methylation of functional lncRNAs has been widely reported in carcinogenesis and progression of CRC; however, the methylation status of *MEF2C-AS1* and its role remains unclear. In this study, we performed a comprehensive analysis of *MEF2C-AS1* methylation status and its expression changes in comparisons of lesion tissues and normal tissues among patients during colorectal carcinogenesis. Firstly, we found high methylation and low expression of *MEF2C-AS1* in CRC tissues by online public databases. Then, the *MEF2C-AS1* hypermethylation and downregulation were found among our patients at all stages of colorectal carcinogenesis as early as in NAAs, and significant differences in methylation levels in lesion tissues were found between AAs and NAAs, CRCs and NAAs. Moreover, patients with *MEF2C-AS1* hypermethylation were found to be more likely to suffer CRC-specific death. Lastly, we confirmed the reduction of *MEF2C-AS1* expression caused by its promoter hypermethylation and also found that its overexpression could suppress proliferation, migration, and invasion in CRC cells.

It is noteworthy that promoter methylation plays an important regulatory role in gene expression [30–32]. Although previous studies have reported that *MEF2C-AS1* was downregulated in DGC [27] and CC [28] lesion tissues, and BC [29] cells, the current study is the first that systematically described the *MEF2C-AS1* methylation status and its influence on expression. Hypermethylation and lower expression were found in 6 cancers including BLCA, BRCA, CESC, UCEC, COAD, and READ. However, for the other 4 cancers including DLBC, OV, TGCT, and UCS, though lower expression levels were found in the comparisons of lesion tissues versus normal tissues, its methylation change and correlation between methylation and expression could not be examined due to the unavailability of methylation data of normal tissues. Further exploration might be performed in future studies.

Promoter hypermethylation is identified to play an important role during tumorigenesis through silencing the gene expression of tumor suppressor genes [33, 34]. Based on a genome-wide screening performed by Li Y et al., aberrant hypermethylation of *α-internexin* gene promoter was identified in both colon adenoma and cancer, and the findings were validated in biospecimens of 30 normal colons, 37 adenomas, and 30 colon cancers using quantitative methylation-specific PCR, and the silenced *α-internexin* expression by epigenetic alteration was confirmed in CRC cells [35]. Using 450 K data and RNA-Seq expression data in public databases, we found the higher methylation and lower expression of *MEF2C-AS1* in the comparisons of colon and rectum adenocarcinoma lesion tissues versus normal tissues. Then, the study was extended to our in-house samples, and we further validated the hypermethylation of *MEF2C-AS1* promoter in CRC and its precursor lesions step-by-step, followed by corresponding downregulation of *MEF2C-AS1*. Additional demethylation assay verified the regulatory role of promoter hypermethylation of *MEF2C-AS1* on its expression in vitro. Taken together, our results demonstrated that *MEF2C-AS1* hypermethylation might be an early driven event during colorectal carcinogenesis.

Colorectal adenoma could be divided into NAA and AA according to the histology clinically, and AA has more advanced features than NAA. The detection and treatment of AA were important for the early prevention of CRC [36]. At present, there are few studies revealing the differences between AA and NAA at the epigenetic level. Our identification that the hypermethylation of *MEF2C-AS1* occurred in colorectal lesions at all stages of colorectal carcinogenesis provided the potential application possibility for this methylation marker in early cancer detection. Furthermore, significant differences in methylation of lesion tissues were found between AAs and NAAs, CRCs and NAAs, but not between CRCs and AAs, indicating the inheritance and development of epigenetic alteration from NAA, to AA, and to CRC. Meanwhile, differences in expression levels were found in the comparison of CRCs and NAAs. Collectively, our findings provide epigenetic evidence for the potential of being an effective biomarker which might be applied in early diagnosis and treatment practice to detect advanced neoplasia including AA and CRC.

The prognostic significance of hypermethylated lncRNA genes has been well proved in various human cancers. For instance, promoter hypermethylation of the lncRNA *PLUT* was significantly associated with shorter relapse-free survival of lung adenocarcinoma [37], and hypermethylated *ZNF667-AS1* was correlated with ESCC patients’ survival [38]. In our study, a reverse association was found between hypermethylation of *MEF2C-AS1* and DSS in CRC patients, revealing the value in developing predictive prognostic models integrating molecular biomarkers with age, TNM stage, or other clinical characteristics [39]. Mechanically, the impact of *MEF2C-AS1* methylation on DSS might be explained by its posttranscriptional regulatory role through a competing endogenous RNA network or cis-activating transcription of near coding genes [40, 41]. In general, our findings suggest the potential that *MEF2C-AS1* hypermethylation might act as a predictor of CRC prognosis.

The tumor suppressor role of *MEF2C-AS1* expression in cancer initiation and progression has been reported in several cancers. Briefly, knock-down of *MEF2C-AS1* promoted aggressive tumor behaviors by reducing the protein levels of *FAT3*, *NTN1*, and *LYVE1* which were related to proliferation and invasion in GC cell lines [27]. Wang X et al. [28] have found that *MEF2C-AS1* played a suppressor role in CC via suppressing miR-592 by targeting RSPO1. Similarly, *MEF2C-AS1* was reported to inhibit proliferation, migration, and invasion of BC cells by targeting miR-3646 downregulation which might be related to the inhibition of CDK1 and MMP-2 protein expression [29]. In our study, we observed that *MEF2C-AS1* overexpression significantly inhibited cell proliferation, migration, and invasion of RKO cells, revealing its potential tumor-suppressing role in colorectal carcinogenesis. Further comprehensive bioinformatics analysis revealed that 3 miRNAs were upregulated by *MEF2C-AS1* downregulation, resulting in the downregulation of 10 mRNAs in CRC. Among them, hsa-miR-429 has been proved to act as an important oncogenic miRNA and be involved in the regulation of several cellular processes contributing to the progression and metastasis of CRC via targeting *SOX2* [42]. The underlying mechanism of whether *MEF2C-AS1* suppresses cell proliferation, migration, and invasion through the ceRNA regulatory pathway needs future exploration.

Our study has several advantages. The consistency of our results and those from online databases could summarize the stability and repeatability of *MEF2C-AS1* hypermethylation status in CRC. Comprehensive analysis of *MEF2C-AS1* methylation status along with expression changes in patients at all stages of colorectal carcinogenesis presents a great value in the CRC etiology at the epigenetic level. This study also has a few limitations. Firstly, the number of patients with colorectal adenoma was limited in the confirmation step. Secondly, samples used for methylation assessment were local tissues in this study. Evidence among other samples like blood and stool might further provide supplementary confirmation for our results. Lastly, the underlying molecular mechanism of hypermethylated *MEF2C-AS1* during CRC development was not fully elucidated, and in vivo and in vitro experiments are necessary for further study.

## Conclusions

In summary, this is the first study to explore the methylation status of *MEF2C-AS1* and its expression in patients at all stages during colorectal carcinogenesis, revealing promoter hypermethylation and low expression in lesion tissues when compared with adjacent normal tissues. The findings reveal that hypermethylation of *MEF2C-AS1* promoter might be an early driven event increasing sequentially in the process of malignant transformation from non-advanced adenoma, to advanced adenoma, and to carcinoma. It is suggested that *MEF2C-AS1* hypermethylation might serve as a promising prognostic biomarker for CRC survival. Moreover, our study also confirms the tumor-suppressing role of *MEF2C-AS1* in colorectal carcinogenesis.

## Materials and methods

### Study design

**Fig. 8:**
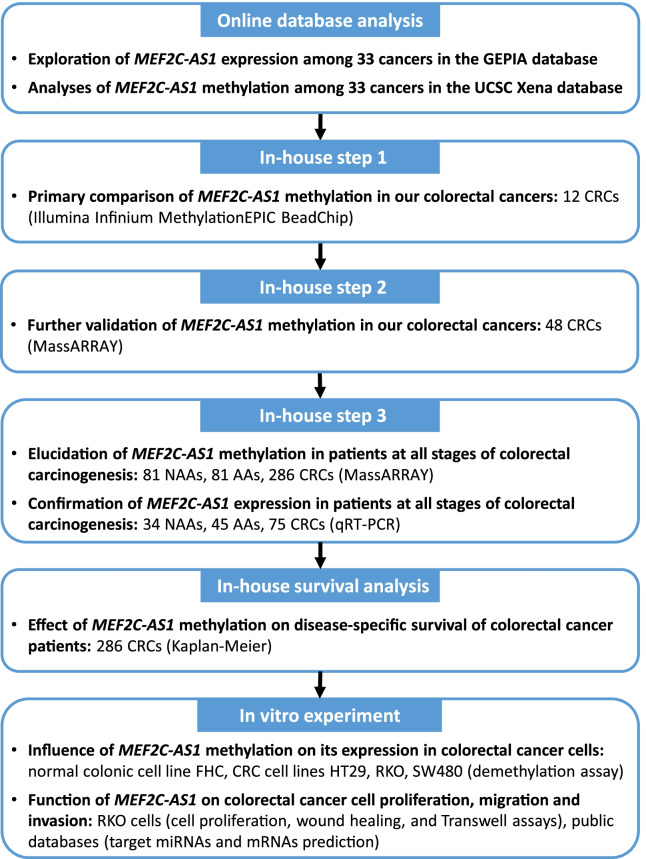
Overall flowchart of the current study. AA, advanced adenoma; CRC, colorectal cancer; NAA, non-advanced adenoma; qRT-PCR, quantitative reverse transcription-polymerase chain reaction

Figure 8 shows the overall flowchart of this study. Firstly, we systematically analyzed the *MEF2C-AS1* expression and methylation status among 31 cancer types using available data based on the GEPIA and UCSC Xena databases. Then, three independent steps were conducted in our in-house samples. In in-house step 1, the methylation status of *MEF2C-AS1* promoter was primarily compared between lesion tissues and adjacent normal tissues using 850 K array scan data from 12 CRCs. In in-house step 2, the *MEF2C-AS1* methylation status was further measured among additional 48 CRCs using the MassARRAY method to technically validate the above array-based findings. In in-house step 3, to clarify the methylation status and corresponding expression changes of *MEF2C-AS1* among patients at all stages during colorectal carcinogenesis, its methylation levels were further elucidated in a larger sample including additional 81 NAAs, 81 AAs, and 286 CRCs also using the MassARRAY method, and its expression levels were confirmed among 34 NAAs, 45 AAs, and 75 CRCs using qRT-PCR. Furthermore, survival analysis was performed in 286 CRCs by the Kaplan–Meier method. In addition, a demethylation assay was used to assess the influence of *MEF2C-AS1* methylation on its expression in CRC cells. And cell proliferation, wound healing and transwell assays in vitro and bioinformatics analysis were performed to evaluate the functional role of *MEF2C-AS1* in colorectal carcinogenesis.

### Patients and samples

The basic characteristics of our own patients are presented in Table 3. CRC patients were collected from Shaoxing People's Hospital between January 2015 and July 2018. Patients with colorectal adenoma have been enrolled from a large-scale population-based screening program for early detection and treatment of colorectal cancer in Jiashan County, Zhejiang Province, China [43]. All patients were ethnic Han Chinese from Zhejiang Province and pathologically confirmed, with no familial adenomatous polyposis (FAP), no previous history of CRC, and none had received any preoperative anticancer treatment. All CRC patients were histologically confirmed with adenocarcinoma. For colorectal adenoma, AA is defined as adenoma ≥ 10 mm in diameter, and/or with high-grade dysplasia or villous or tubulovillous histology, and NAA is defined as adenoma < 10 mm without advanced histology according to guidelines [44]. For each patient, both lesion tissue and adjacent normal tissue at a distance of at least 5 cm away were taken simultaneously through surgery or colonoscopy. Information on characteristics including age, gender, tumor location, TNM stage [45], and pathological differentiation was also obtained. Patients who participated in the study signed informed consents, and the study was approved by the Medical Ethics Committee of Zhejiang University School of Medicine.

**Table 3:** Basic characteristics of in-house patients in three independent steps

Characteristic	In-house step 1	In-house step 2	In-house step 3					
			Methylation analysis			Expression analysis		
	CRC	CRC	NAA	AA	CRC	NAA	AA	CRC
Total	12	48	81	81	286	34	45	75
Age, mean ± SD	63.5 ± 4.2	62.8 ± 9.3	63.5 ± 6.7	62.8 ± 6.6	64.9 ± 11.8	56.9 ± 9.4	61.0 ± 7.0	64.9 ± 12.1
*Gender*								
Male	7	24	40	41	171	20	30	40
Female	5	24	41	40	115	14	15	35
*Location*								
Colon	6	22	60	59	133	33	45	28
Rectum	6	26	21	22	153	1	0	47
*TNM stage*								
I-II	6	24			143			41
III-IV	6	24			143			34
*Differentiation*								
High	7	24			83			32
Middle	4	23			185			41
Low	0	1			6			0
Missing	1	0			12			2

*AA*, advanced adenoma; *CRC*, colorectal cancer; *NAA*, non-advanced adenoma; *SD*, standard deviation

### Cell culture and treatment

The normal colonic epithelial cell line FHC was cultured in DMEM/F12 medium, and the CRC cell lines HT29, RKO, and SW480 were also cultured in DMEM medium. All the mediums were supplemented with 1% antibiotics and 10% fetal bovine serum (FBS). All cells were cultured in a thermostatic incubator with 5% CO_2_ at 37 °C. For the demethylation assay, the CRC cells (5 × 10^5^/well) were treated with different concentrations (0, 10, 15, 20 µM) of demethylation agent 5-Aza-dC (AdooQ Bioscience, USA) for 24 h. The *MEF2C-AS1* expression levels in different cell lines were measured by qRT-PCR.

### Online database

GEPIA (http://gepia.cancer-pku.cn/) [46] is a newly developed interactive web server for analyzing the RNA-Seq expression data of 9,736 tumor and 8,587 normal samples of 33 cancer types from the TCGA and the Genotype-Tissue Expression (GTEx) projects. The expression data of *MEF2C-AS1* were presented on the scale of log_2_(TPM + 1).

DNA methylation data of 33 cancers, measured experimentally using the Illumina Infinium HumanMethylation450 BeadChip (450 K array), were downloaded from the UCSC Xena database (https://xena.ucsc.edu/) [47]. DNA methylation values, described as beta values (β), are continuous variables between 0 and 1, representing the ratio of the intensity of the methylated bead type to the combined locus intensity. Four CpG sites (cg04694437, cg10571951, cg12621171, and cg18109838) were identified in *MEF2C-AS1* promoter, and their mean level was considered as its methylation.

### Genome-wide DNA extraction and bisulfite conversion

Genomic DNA was extracted from fresh-frozen tissue samples using the DNA Tissue Kit (TianLong Biotech, Xi’an, China), and the concentration and purity of DNA were measured using NanoDrop 2000 spectrophotometer (Thermo Scientific, Wilmington, DE, USA) following the manufacturer’s instructions. The EZ Methylation Gold Kit (Zymo Research, Irvine, CA, USA) was used to conduct bisulfite treatment on genomic DNA (500 ng).

### DNA methylation assessment

*MEF2C-AS1* methylation status of 12 pairs of lesion tissues and adjacent normal tissues of CRC patients were assessed using 850 K array as previously described [48]. Six CpG sites (cg04694437, cg08966485, cg15297153, cg10571951, cg12621171, and cg18109838) in the *MEF2C-AS1* promoter were identified according to the annotation mapped against the human reference version GRCh37/hg19. The mean level of six CpG sites was considered as the methylation level of *MEF2C-AS1*.

The validation of *MEF2C-AS1* methylation was performed on the Sequenom MassARRAY platform (Sequenom, San Diego, CA, USA). The primers (forward: 5’-aggaagagagAGTAGGAGGTAGGTTTTGGGTTTTT-3’; reverse: 5’-cagtaatacgactcactatagggagaaggctCCCTCTTACTCTCCCAAATTTACA-3’) were designed using EpiDesigner (http://epidesigner.com). The tested sequence (chr5:88,185,329–88,185,810, GRCh37/hg19) contained 26 CpG sites, and sites outside of the mass spectrometry analytical window were filtered out of the methylation measurement (Additional file 1: Fig. S3). The MassARRAY Compact MALDI-TOF system (Sequenom, BioMiao Biological Technology, Beijing, China) was used to collect the mass spectra, and the methylation proportions were generated by EpiTYPER software (Sequenom, San Diego, CA). Methylation level was expressed as beta value (β), which was the ratio of methylated intensity to the sum of methylated intensity and unmethylated intensity with a range from 0 (completely unmethylated) to 1 (completely methylated). Samples and CpG sites with > 10% missing values were eliminated in the subsequent analysis. The mean level of all individual CpG sites was considered as the *MEF2C-AS1* methylation level*.*

### RNA isolation and qRT-PCR

Total RNA was extracted using TRIzol reagent (Invitrogen, Carlsbad, CA, USA) following the manufacturer’s instructions. The cDNA was synthesized from 1 µg of total RNA using Moloney Murine Leukemia Virus reverse transcriptase (Takara, Otsu, Shiga, Japan). The qRT-PCR was performed on LightCycler-480 system (Roche, Mannheim, Germany) with SYBR Green protocol. *β-actin* was used as an internal control. Relative expression levels were calculated by 2^−ΔΔCT^ or 2^−ΔCT^ method. The primer sequences were as follows: 5’-GTGGCCGAGGACTTTGATTG-3’ (forward) and 5’-CCTGTAACAACGCATCTCATATT-3’ (reverse) for *β-actin*, and 5’-GCTCCTAGGTATGGGTGGGA-3’ (forward) and 5’-TTTGTTGTGTGGTGCGACAG-3’ (reverse) for *MEF2C-AS1*.

### Cell transfection

The pLV3 vector and pLV3-MEF2C-AS1 plasmid were constructed by Shanghai Hewu Biotechnology Co., Ltd. (Shanghai, China). The cells were cultured overnight and transfected by these two plasmids with the Lipofectamine™ 2000 Transfection Reagent (Invitrogen, USA) following the manufacturer’s protocol. The *MEF2C-AS1* expression was measured by qRT-PCR 24 h later.

### Cell proliferation assay

The direct cell count method was used to examine cell proliferation. Briefly, 1.2 × 10^5^ cells/well were seeded in 12-well plates, and cells were transfected with control (pLV3 vector) or pLV3-MEF2C-AS1 plasmids after 24 h, respectively. Cells were collected at 24 h and 48 h after transfection and counted using a CytoSMART Exact FL Cell Counter (CytoSMART Technologies, Netherlands).

### Wound healing and transwell assays

The transfected RKO cells (pLV3 vector and pLV3-MEF2C-AS1 plasmid) were seeded in 6-well plates. A wound healing assay was used to assess the cell migration. The wound margins were photographed at 0 h and 24 h after scratching the cells with a 200 µl sterile pipette tip. Cell migration and invasion assays were performed using 8-µm transwell chambers (BIOFIL, China). For migration assay, the cells (2 × 10^5^/well) were plated in serum-free medium onto the upper chamber and a 500 µl medium containing 30% FBS was added to the lower chamber. After incubation for 72 h, the cells were fixed with 4% paraformaldehyde and stained with 0.1% crystal violet. The migrated cells on the lower surface were photographed under an inverted microscope (Olympus CKX53, Japan) and counted. For invasion assay (3 × 10^5^ cells/well), the upper chamber was precoated with Matrigel (Corning, USA), and all other processes were the same.

### Target miRNA and mRNA prediction

The differentially expressed miRNAs and mRNAs were analyzed using data from the TCGA and UCSC Xena databases. The miRNAs targeted by MEF2C-AS1 were predicted using the miRcode database (http://www.mircode.org/) [49], and then the target mRNAs regulated by miRNAs were predicted by the combinative analysis using the miRDB (http://mirdb.org/) [50], miRTarBase (https://mirtarbase.cuhk.edu.cn/) [51], and TargetScan (http://www.targetscan.org/) databases [52].

### Statistical analysis

The continuous variable was expressed as the mean ± standard deviation (SD), and the categorical variable was expressed as the frequency. Paired Student’s *t* test and Wilcoxon test were used to compare the differences between paired groups. The Kruskal–Wallis test and one-way analysis of variance (ANOVA) followed by the post hoc Dunn’s and Dunnett’s tests were used to compare the differences among multiple groups. Survival time was calculated from the date of diagnosis to the death date or the end date of uniform follow-up. CRC patients were further divided into groups of high and low methylation using maxstat statistics in the R package “survminer”. The Kaplan–Meier method and log-rank test were used to compare the DSS of CRC patients in different groups. All statistical analyses were performed in R software (version 3.6.1). A two-tailed *p* value less than 0.05 was considered statistically significant.

## Supplementary Information

**Additional file 1**: **Fig. S1**. Nonsignificant difference in MEF2C-AS1 expression between lesion tissues and normal tissues among 21 cancers by the GEPIA database. Every dot represents the expression level for a tissue sample. Box plot in red or gray represents the distribution of expression level. Expression level is presented in log_2_(TPM+1) scale. TPM, Transcripts Per Million. ns *p* > 0.05. **Fig. S2**. Comparisons of MEF2C-AS1 methylation between lesion tissues and normal tissues among 21 cancers by the UCSC Xena database. Violin plot in blue or orange represents the distribution of methylation level. * *p* < 0.05, ns *p* > 0.05. **Fig. S3**. The amplified sequence of MEF2C-AS1 was used for the methylation measurement by the MassARRAY method. CpG sites in capital letters were measured, and CpG_1 and CpG_25 correspond to cg10571951 and cg12621171, respectively. CpG sites highlighted in yellow were successfully measured, and those in gray were filtered for methylation analysis. **Table S1**. The putative predicted target miRNAs and mRNAs of MEF2C-AS1 in CRC.

## Data Availability

The datasets used and/or analyzed during the current study are available from the corresponding author on reasonable requests. RNA-Seq expression data and 450 K array methylation data for 33 cancers could be obtained from the GEPIA (http://gepia.cancer-pku.cn/), UCSC Xena (https://xena.ucsc.edu/), and TCGA (https://portal.gdc.cancer.gov/) databases, respectively. Prediction of target miRNAs and mRNAs was performed by the miRcode (http://www.mircode.org/), miRDB (http://mirdb.org/), miRTarBase (https://mirtarbase.cuhk.edu.cn/), and TargetScan (http://www.targetscan.org/) databases.

## Abbreviations

450 K array: Illumina HumanMethylation450 BeadChip
5-Aza-dC: 5-Aza-2’-deoxycytidine
850 K array: Illumina MethylationEPIC BeadChip
AA: Advanced adenoma
ANOVA: Analysis of variance
BC: Breast cancer
CC: Cervical cancer
ceRNA: Competitive endogenous RNA
CpG: Cytosine–guanine dinucleotide
CRC: Colorectal cancer
CSCC: Cervical squamous cell carcinoma
DGC: Diffuse gastric cancer
DSS: Disease-specific survival
ESCC: Esophageal squamous cell carcinoma
FAP: Familial adenomatous polyposis
FBS: Fetal bovine serum
GTEx: Genotype-Tissue Expression
lncRNA: Long noncoding RNA
MEF2C-AS1: MEF2C antisense RNA 1
NAA: Non-advanced adenoma
OS: Osteosarcoma
PC: Pancreatic cancer
qRT-PCR: Quantitative reverse transcription-polymerase chain reaction
RNA-Seq: RNA sequencing
SD: Standard deviation
TCGA: The Cancer Genome Atlas
TPM: Transcripts per million
