# Supplementary material for: Hypermethylation of tumor suppressor lncRNA MEF2C-AS1 frequently happened in patients at all stages of colorectal carcinogenesis
Source: Clin Epigenetics. 2022 Sep 5;14:111. doi: 10.1186/s13148-022-01328-1 (PMC9446566; doi:10.1186/s13148-022-01328-1)
Supplement: Supplementary file 1 — Additional file 1: Fig. S1. Nonsignificant difference in MEF2C-AS1 expression between lesion tissues and normal tissues among 21 cancers by the GEPIA database. Every dot represents the expression level for a tissue sample. Box plot in red or gray represents the distribution of expression level. Expression level is presented in log2(TPM+1) scale. TPM, Transcripts Per Million. ns p > 0.05. Fig. S2. Comparisons of MEF2C-AS1 methylation between lesion tissues and normal tissues among 21 cancers by the UCSC Xena database. Violin plot in blue or orange represents the distribution of methylation level. * p < 0.05, ns p > 0.05. Fig. S3. The amplified sequence of MEF2C-AS1 was used for the methylation measurement by the MassARRAY method. CpG sites in capital letters were measured, and CpG_1 and CpG_25 correspond to cg10571951 and cg12621171, respectively. CpG sites highlighted in yellow were successfully measured, and those in gray were filtered for methylation analysis. Table S1. The putative predicted target miRNAs and mRNAs of MEF2C-AS1 in CRC. [file 13148_2022_1328_MOESM1_ESM.docx]

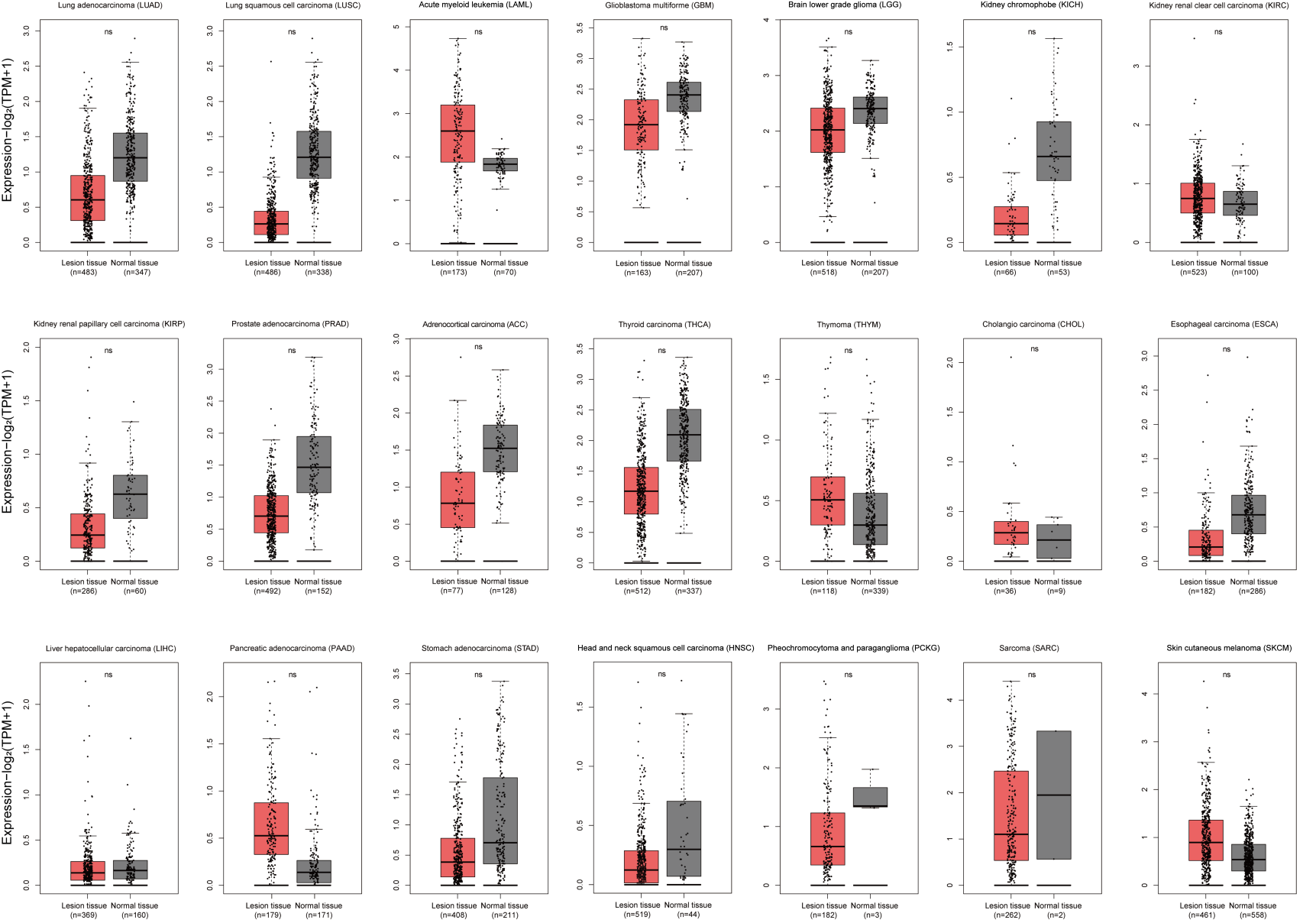


**Fig. S1** Nonsignificant difference in *MEF2C-AS1* expression between lesion tissues and normal tissues among 21 cancers by the GEPIA database. Every dot represents the expression level for a tissue sample. Box plot in red or gray represents the distribution of expression level. Expression level is presented in log_2_(TPM+1) scale. TPM, Transcripts Per Million. ns *p* > 0.05

**
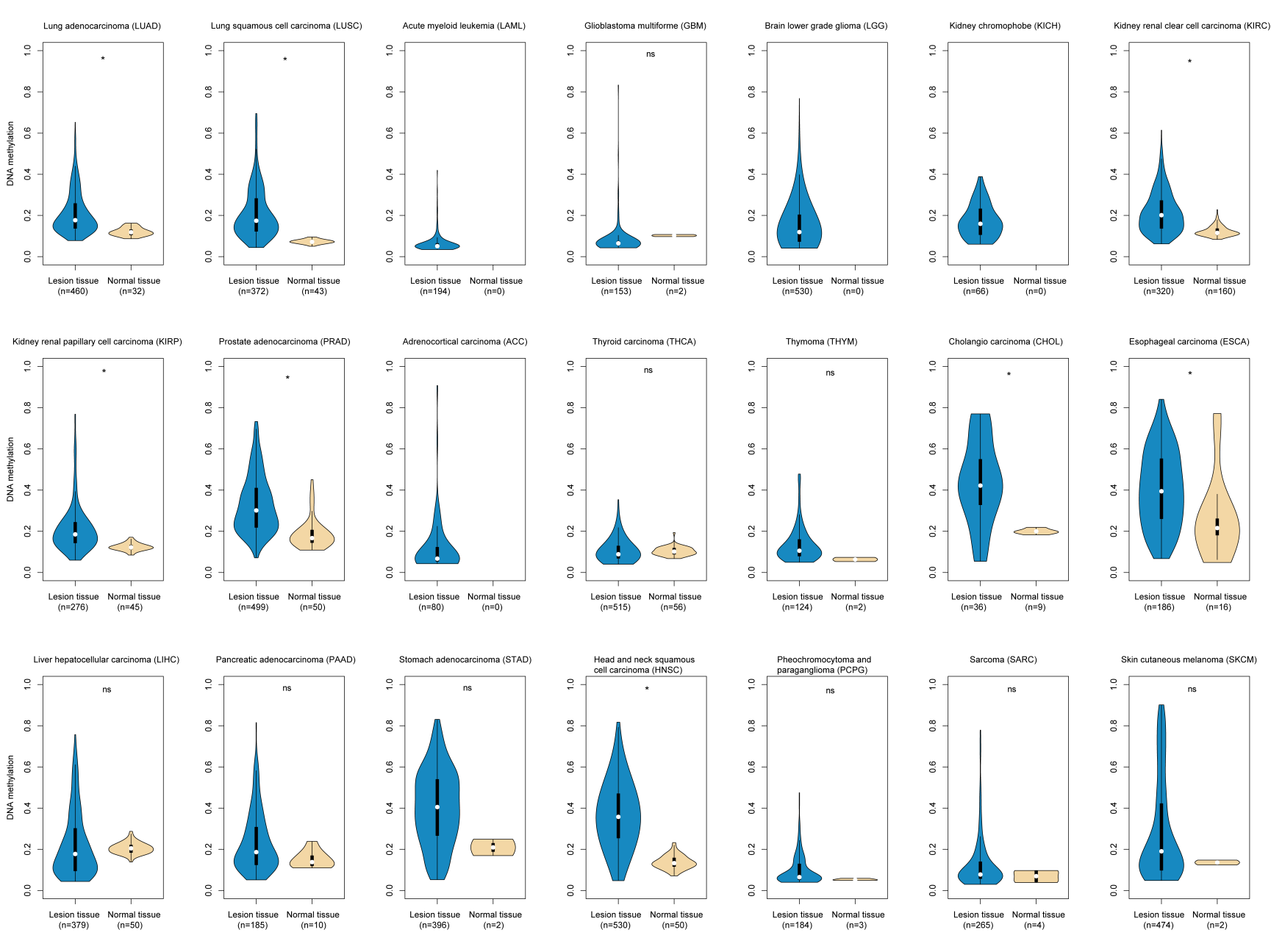
**

**Fig. S2** Comparisons of *MEF2C-AS1* methylation between lesion tissues and normal tissues among 21 cancers by the UCSC Xena database. Violin plot in blue or orange represents the distribution of methylation level. * *p* < 0.05, ns *p* > 0.05


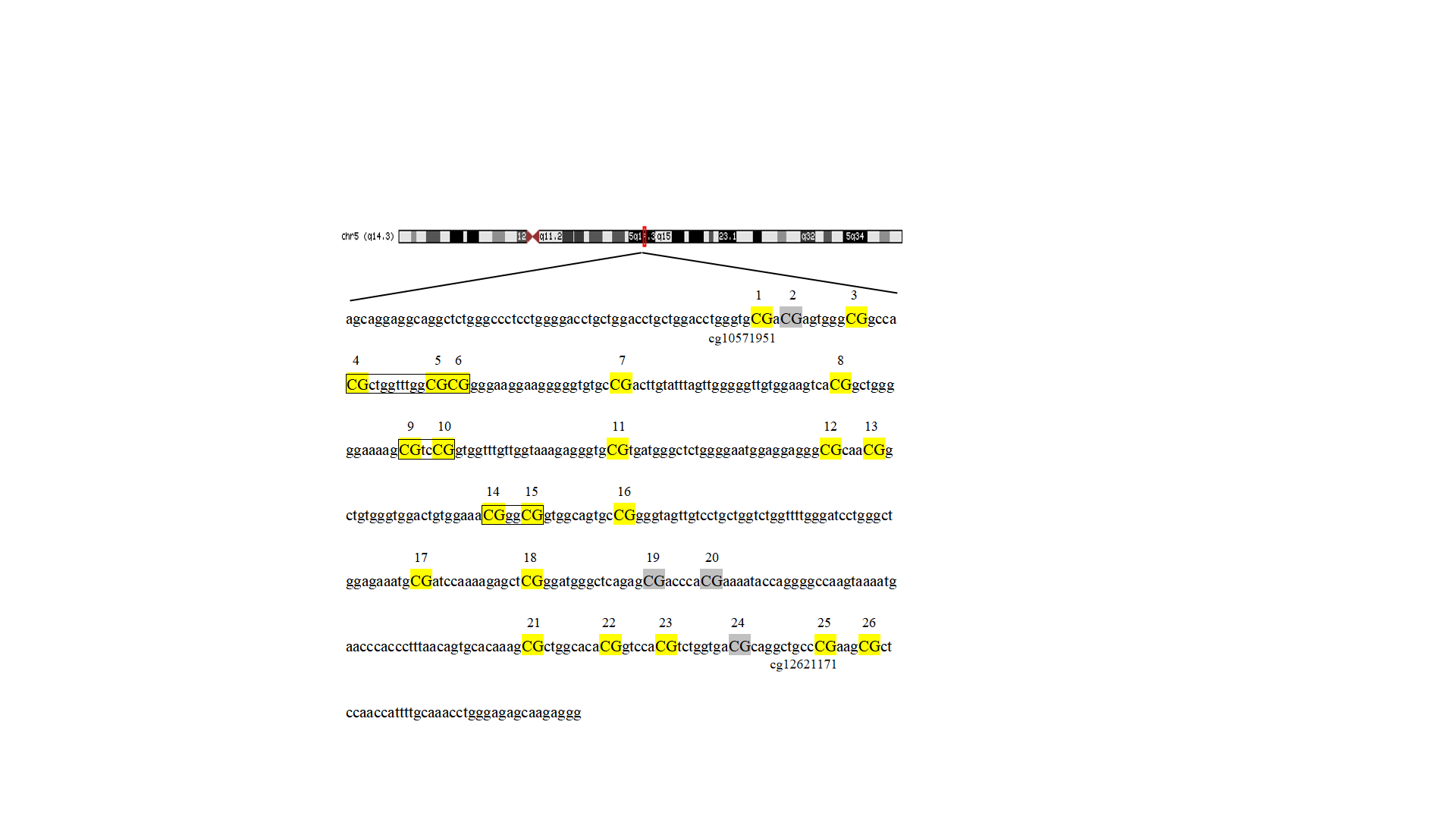


**Fig. S3** The amplified sequence of *MEF2C-AS1* used for the methylation measurement by MassArray method. CpG sites in capital letters were measured, and CpG_1 and CpG_25 correspond to cg10571951 and cg12621171, respectively. CpG sites highlighted in yellow were successfully measured, and those in gray were filtered for methylation analysis

| **Table S1** The putative predicted target miRNAs and mRNAs of *MEF2C-AS1* in CRC | | | | | | | |
| --- | --- | --- | --- | --- | --- | --- | --- |
| LncRNA | |  | Mature miRNA | |  | Protein coding gene | |
| Name | Expression |  | Name | Expression |  | Name | Expression |
| *MEF2C-AS1* | Down |  | hsa-miR-17-5p | Up |  | *KCNB1*, *CFL2*, *FAM129A*, *CLIP4*, *CYBRD1*, *SLC16A9*, *TMEM100* | Down |
|  |  |  | hsa-miR-24-3p | Up |  | *BVES*, *TRPM6* | Down |
|  |  |  | hsa-miR-429 | Up |  | *ZEB1* | Down |
